# Effect of mesenchymal stem cells on small intestinal injury in a rat model of acute necrotizing pancreatitis

**DOI:** 10.1186/s13287-017-0471-z

**Published:** 2017-01-23

**Authors:** Fengchun Lu, Feng Wang, Zhiyao Chen, Heguang Huang

**Affiliations:** General Surgery Department, Fujian Medical University Union Hospital, Fuzhou, 350001 China

**Keywords:** Mesenchymal stem cells, Acute necrotizing pancreatitis, Intestinal injury, Capillary leakage, Aquaporin 1

## Abstract

**Background:**

Acute necrotizing pancreatitis (ANP) is often complicated by multiple organ failure. The small intestine is frequently damaged during ANP. Capillary leakage in multiple organs during ANP is one of the most important causes of multiple organ dysfunction. Damage to the capillary endothelial barrier and impaired water transportation could lead to capillary leakage in ANP.

**Methods:**

Sprague–Dawley (SD) rats were randomized into a control group, the ANP group, the culture media-treated group, or the bone marrow-derived mesenchymal stem cell (BMSC)-treated group (30 rats in each group). Ten rats in each group were sacrificed at 6, 12, and 24 h after induction of experimental models. Serum, ascites, pancreatic, and small intestinal samples were collected. The levels of serum and ascites albumin and amylases were measured, pancreatic histology was assessed, and the connection changes between vessel endothelial cells were evaluated using scanning electron microscopy (SEM). Capillary leakage in small intestinal tissue was observed visually by tracking fluorescein isothiocyanate (FITC)-albumin, and was measured by the Evans blue extravasation method. The location and expression of aquaporin 1 (AQP1) in the small intestine was analyzed using immunohistochemistry, real-time polymerase chain reaction (PCR), and Western blot.

**Results:**

The outcomes showed that the level of serum and ascites amylase is elevated. Conversely, the level of serum albumin is decreased while ascites albumin is elevated. There is damage to pancreatic tissue, and the small intestinal capillary endothelial barrier was aggravated. Furthermore, the expression of AQP1 was reduced significantly after induced ANP. Following treatment with MSCs, the elevation of amylase and the decrease of serum albumin were inhibited, the damage to pancreatic tissue and the level of small intestinal capillary leakage was alleviated, and the downregulation of AQP1 was reversed.

**Conclusions:**

In conclusion, MSC therapy could alleviate small intestinal injury in rats with ANP, the mechanism of which might be related to reduction of damage to the small intestinal capillary endothelial barrier, and increased expression of AQP1 in the small intestine.

## Background

Acute necrotizing pancreatitis (ANP) is a challenging disease with high morbidity and mortality, often complicated by multiple organ failure. Two phases of ANP are observed in the clinic: the early toxico-enzymatic phase and the later septic phase [1]. Capillary leakage occurs in the early phase of ANP. Capillary leakage in multiple organs during ANP is one of the most important causes of multiple organ dysfunction. The small intestine is frequently damaged during ANP. Intestinal edema, abundant amylase and bloody ascites, and intestinal barrier functional disturbances can be found in ANP. The damage to the capillary endothelial barrier and the function of water transportation could lead to capillary leakage in ANP.

Aquaporins (AQPs) form the water channels that are responsible for the water permeability of some biological membranes [2]. AQP1 is the first member of the AQP family to be identified in erythrocytes [3]. In the small intestine, AQP1 is widely distributed on the capillary or microvascular endothelium of lymphatic cells [4–7]. Studies show that the abnormality of AQP1 is a main contributor to many diseases [2, 6]. The expression of AQP1 is significantly reduced in a murine model of lipopolysaccharide (LPS)-induced acute lung injury [8], and in the pancreas, lung, and intestinal tissue of rats with ANP [9].

Mesenchymal stem cells (MSCs) have the capability to regulate the immune system and regenerate damaged tissue, making them good candidates for cell-based therapy [10–13]. MSCs reduced inflammation and damage to pancreatic tissue in a rat model of acute pancreatitis [14]. MSC treatment could increase the speed of recovery and restore function to the small intestine in a mouse model of radiation-induced gastrointestinal tract damage [15]. MSCs could reduce vascular endothelium injury, and decrease endothelial permeability induced by LPS [16]. Whether MSCs have protective effects in small intestinal capillary leakage in ANP has not yet been studied.

In this study, we examined the changes of the small intestinal capillary endothelial barrier and AQP1 expression in the rat model of ANP, and explored whether MSCs have a protective effect on small intestinal injury induced by ANP through their impact on the capillary endothelial barrier and expression of AQP1 in the small intestine.

## Methods

### Ethics

All animal experimental procedures were approved by the Experimental Animals Committee of Fujian Medical University. All animals received humane care in compliance with the Guide for the Care and Use of Laboratory Animals (NIH publication No.85-23, National Academy Press, Washington, DC, USA, revised 1996).

### Experimental animals and grouping

Clean adult male Sprague–Dawley (SD) rats, weight 200–250 g, were provided by Shanghai SLAC Laboratory Animal Co. Ltd., and adaptively fed for a week in a room with temperature maintained at 20 ± 2 °C. Experimental animals were treated according to ethical guidelines and standards. Rats (*n* = 120) were randomly divided into a control group, culture media-treated group, ANP group, and MSC-treated group (*n* = 30 per group)*.* Observations were made at three time points (6 h, 12 h, and 24 h; *n* = 10 for each) and the rats were sacrificed at each time point for sample collection.

### Culture, identification, and labeling of MSCs

MSCs were isolated from the bone marrow of 1-month-old male SD rats by the differential adherence method [17–19]. In brief, the tibia and femur of SD rats were separated under sterile conditions to expose the bone marrow cavity which was flushed with Dulbecco’s modified Eagle’s medium (DMEM; GE Healthcare Life Sciences, Logan, UT, USA) culture media. The bone marrow filtrate was collected and centrifuged (1500 rpm, 3 min). The cells were resuspended in DMEM supplemented with 10% fetal calf serum (FCS) and 1% penicillin/streptomycin, and then inoculated in a 25-mL culture flask at the concentration of 5 × 10^7^/mL and incubated at 37 °C and 5% CO_2_. Two days after plating, the dishes were washed three times with 10% FCS-DMEM to remove non-adherent hematopoietic cells. Subsequently, the medium was changed every 2–3 days and cells were maintained until reaching 80–90% confluence. Confluent cells were dissociated using EDTA-Trypsin (Invitrogen, San Diego, CA, USA). They were cultured for multiple generations and purified by dissociation. MSCs in the third generation were acquired for further experiments. Expression of surface markers CD29, CD45, CD90 (Biolegend, San Diego, CA, USA), and CD34 (Santa Cruz Biotechnology, Santa Cruz, CA, USA) on MSCs was confirmed by flow cytometry analysis (FACS; Canto, Becton Dickinson, San Jose, CA, USA). MSCs were labeled using the cell tracker CM-Dil (Molecular Probes Inc., USA) according to the manufacturer’s instructions. MSCs in the third generation with a concentration of 1 × 10^6^ cells/mL were cultured in 5 μL CM-Dil labeling solution (2 mg CM-Dil/mL). CM-Dil cell suspensions were incubated for 5 min at 37 °C and then for 15 min at 4 °C. After labeling, cells were washed two times with phosphate-buffered saline (PBS) at 1000 rpm for 5 min at 25 °C and resuspended in fresh medium.

### Establishment of the ANP animal model

The method used to create the rat model of ANP was as described in previous studies [20–22]. Rats were fasted for 12 h before the experiments, but allowed free access to water. After anesthesia with 10% chloral hydrate (0.3 mL/100 g body weight; Bio Basic, Markham, ON, Canada), an incision was made in the abdominal midline under aseptic conditions. The bile duct near the portal vein was clipped with a small bulldog clamp. Using a surgical microscope (magnification 10×), a polyethylene catheter (0.45 mm in diameter) was inserted into the biliopancreatic duct where it enters the duodenum, and was also clipped with a small bulldog clamp. Then 5% sodium taurocholate solution (0.1 mL/100 g body weight; Inalco Spa, Milano, Italy) was injected into the biliopancreatic duct at a uniform speed of 0.04 mL/min with a microinfusion pump, and the small bulldog clamp was removed 10 min later. The duodenum was replaced and the abdomen was closed. For rats in the control group, the abdomen was closed after the pancreas and duodenum were maneuvered as they were with the ANP model. Following surgery, the rats were restricted from water and food intake and normal saline was given via subcutaneous injection in the back (4 mL/100 g body weight; every 6 h).

### Treatment with MSCs

Previous studies showed that MSCs transplanted via tail vein injection might reside in the small intestine, limiting gastrointestinal tract damage [15, 23, 24]. ANP-induced animals were injected with 1 mL of MSCs (about 1 × 10^6^/mL) via the tail vein at 0 h, and sacrificed at 6 h for the MSC-treated group 6-h time point. ANP-induced animals were injected with 1 mL of MSCs (about 1 × 10^6^/mL) via the tail vein at 0 h and 6 h, and sacrificed at 12 h for the MSC-treated group 12-h time point. ANP-induced animals were injected with 1 mL of MSCs (about 1 × 10^6^/mL) via the tail vein at 0 h, 6 h, and 12 h, and sacrificed at 24 h for the MSC-treated group 24-h time point. The 0 h means the moment when the abdomen was closed. The rats of the control group and ANP group were injected with 1 mL normal saline via the tail vein at the same time points as the MSC-treated group. The rats in the culture media-treated group were injected with 1 mL of culture media (DMEM supplemented with 10% FCS and 1% penicillin/streptomycin) via the tail vein at the same time points as the MSC-treated group. After treatment with MSCs, small intestine samples were collected at each time point. Frozen small intestinal samples were cut into 10-μm sections and observed under a confocal microscope (TCS SP5, Leica, Germany) and pictures were taken.

### Biochemical tests of blood and ascites

At each time point, blood and ascites samples were collected and measured. They were centrifuged at 3000 rpm at 5 °C for 10 min. The serum and supernatant of ascites were collected and stored at −80 °C until analyzed. Amylase and albumin levels in serum and ascitic fluid were analyzed using an automatic biochemical detection instrument (LX20; Beckman Inc., USA).

### Small intestine capillary leakage measurement using the Evans blue method

To evaluate small intestinal capillary leakage we prepared another nine groups of experimental animals following the same experimental procedures previously described [25–27]. Thirty minutes before the rats were sacrificed, 5% Evans blue (20 mg/kg body weight) was injected into the femoral vein. Thirty minutes later, the chest was accessed through the diaphragm, a catheter was put into the left ventricle, and the right atrium was opened. Cold PBS was perfused into the heart with the infusion apparatus under 40 cm water column pressure until the fluid coming out of the right atrium became clear. Next, 300 mg of tissue from the intestine was incubated in 1 mL formaldehyde and homogenized with an ultrasonic cell disruption device. Another 3 mL of formaldehyde was added for preservation. After being maintained in a 37 °C incubator for 48 h, the samples were centrifuged (1000 rpm, 5 min) and supernatant was analyzed at 620 nm and 740 nm in a spectrophotometer (GE Ultrospec 3100 pro, Amersham Biosciences, USA). The correction formula is: E620_corrected_ = E620 – (1.426 × E740 + 0.030). After the Evans blue standard curve was drawn, concentrations of Evans blue in the intestinal tissue were calculated using Curve Expert 1.3 software. Evans blue content was then calculated based on the concentration values and presented as ng/mg.

### Histopathology

The body of the pancreas was harvested and fixed in 10% formalin solution over 24 h, embedded in paraffin, sectioned at 4-μm thickness, and stained with hematoxylin and eosin (H&E). Pathological changes of the pancreas were observed under an optical microscope.

### Scanning electron microscopy

For scanning electron microscopy (SEM), the venules of rat small intestine mesentery were immediately removed, cut along their longitudinal axis to open the lumen, and fixed in 3% glutaraldehyde/1.5% paraformaldehyde/0.1 M CBS, pH 7.4. Then all samples were sequentially fixed in 1% osmium tetroxide solution, dehydrated with a graded series of ethanol, transferred to t-butyl alcohol, and freeze dried with a freeze dryer (ES-2030, Hitachi High-Technologies, Japan). Tissues were coated with gold palladium particles and examined using a scanning electron microscope (JSM-6380LV, JEOL Ltd., Japan).

### Visualization of capillary leakage by tracking of FITC-albumin

Using fluorescein isothiocyanate (FITC)-albumin as a plasma marker, capillary leakage of the intestine was observed visually. Fifteen minutes before the rats were sacrificed 1 mg/0.5 mL FITC-albumin (Sigma, MO, USA) was injected into the femoral vein. After 15 min, the abdomen was opened and the intestinal tissues and small intestinal mesentery were harvested. The intestinal tissues were fixed in 4% paraformaldehyde, and embedded in Tissue-Tek OCT compound (Sakura Finetek USA, Inc.). Frozen sections (10-μm thickness) were cut at −20 °C with a cryomicrotome (Reichert Jung 2800, Leica, Germany). The small intestinal mesentery was placed on glass slides. They were observed under a confocal microscope and pictures were taken.

### Immunohistochemistry staining of AQP1 in the intestine

After being fixed in 4% paraformaldehyde/3% glutaraldehyde/0.1 M PBS for 8–24 h, small intestinal tissues were embedded and sectioned (5-μm thickness). Then the sections were dewaxed and rehydrated, followed by removal of endogenous peroxidase by peroxide and retrieved with citrate antigen. Sections were sequentially blocked with 5% bovine serum albumin, incubated with anti-AQP1 primary antibody (1:200; Abcam, Cambridge, UK) for 2 h at 37 °C, and with secondary antibody (1:100; Abcam, Cambridge, UK) for 20 min at 37 °C. Sites of peroxidase activity were visualized with DAB. Finally, sections were stained with hematoxylin, dehydrated, and cleared with gradient alcohol and xylene, and mounted in a neutral mounting medium.

### Western blotting

Small intestinal tissue was homogenized on ice and 50 μL of 2 × cell lysis buffer containing protease inhibitors (Cell Signaling, USA) was added to each sample. Homogenized samples were mixed and incubated on ice. Next, 50 μL 2 × SDS loading buffer was added. After proteins were separated in SDS PAGE (12% separating gel and 3% spacer gel), they were transferred to a PVDF membrane. The membrane was first incubated with AQP1 antibody with 1:1000 dilution for 2 h at 25 °C (mouse anti-rat AQP1 IgG; Santa Cruz Biotechnology, Inc., USA) and then incubated with secondary antibody with 1:5000 dilution for 1 h at 25 °C (Abcam, USA), according to the manufacturer’s instructions. Densitometric results were analyzed with Quantity One image-analysis software (Bio-Rad, Hercules, CA, USA).

### Real-time PCR

Total cellular RNAs were extracted from the small intestinal tissues with RNAiso™ Plus (TaKaRa, Japan) according to the instruction of the manufacturer. Reverse transcriptions were done with the ReverTra qPCR RT kit (FSQ-101; TOYOBO, Japan); the reaction conditions were 37 °C for 30 min, then 98 °C for 5 min. The 20 μL of real-time polymerase chain reaction (PCR) reaction mixture contained 2 μL cDNA, 10 μL SYBR® Premix Ex Taq™ II (Perfect Real Time, DRR081, TaKaRa), 0.8 μL PCR forward primer, 0.8 μL PCR reverse primer, 0.4 μL ROX reference dye (50×), and 6 μL dH2O. The PCR primers include GAPDH: 5’-TCTTC CAGGA GCGAG ATCCC-3’/5’-AGTGA TGGCA TGGAC TGTGG TCAT-3’ (PCR product, 320 bp), and AQP1: 5’-TCACT TGGCC GAAAT GACCTG-3’/5’-GTCCC ACCCA GAAAA TCCAG T-3’ (PCR product, 280 bp). The PCR was run in a real-time PCR instrument (7500; ABI Co., USA) at 95 °C for 30 s, followed by incubation at 95 °C for 5 s and 60 °C for 31 s, for a total of 40 cycles.

### Statistical analysis

Data are presented as mean ± SE. A two sample *t* test was performed to compare the difference in values between two groups, and one-way analysis of variance (ANOVA) with a post-hoc Fisher’s least-significant-difference test was applied to identify any significant change of value among several groups. All statistical assessments were considered significantly different at a value of *p* < 0.05. Statistical analyses were performed using SPSS 15.0 statistics software (SPSS Inc., Chicago, IL, USA).

## Results

### Culture, identification, and labeling of MSCs, and tracking in the small intestine

**Fig. 1:**
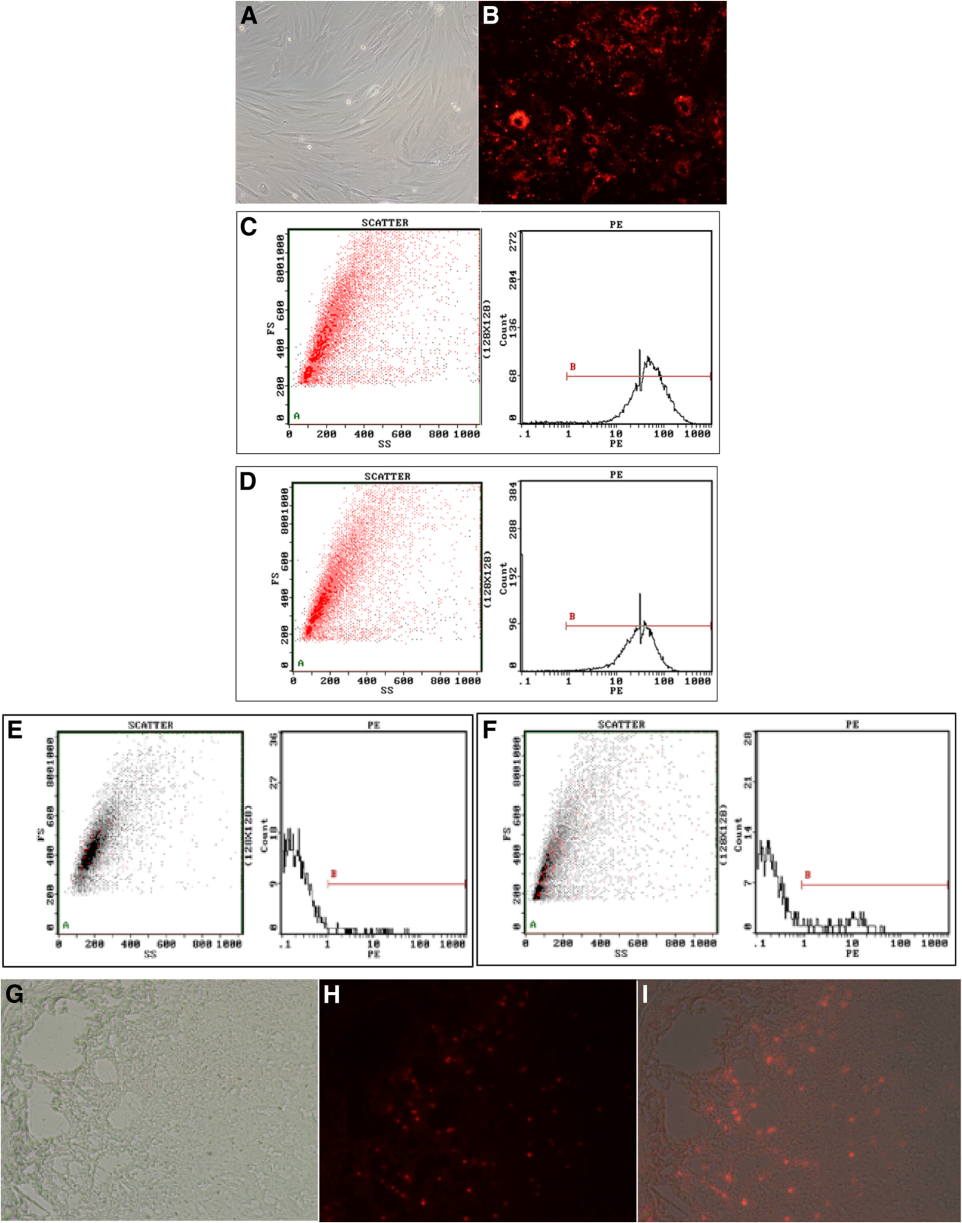
Characterization of MSCs isolated from rat bone marrow and tracking of infused CM-Dil-labeled MSCs in the small intestine. **a** Typical cell morphology of MSCs with adherent growth as spindle-shaped and fibroblast-like, whirl-like distribution at 3 days of the third passage culture (×200). **b** MSCs displayed red fluorescence that was evenly labeled with CM-Dil (×200). **c**–**f** Expression of surface markers of MSCs by flow cytometry. The surface markers of **c** CD29, **d** CD90, **e** CD34, and **f** CD45 were 93.3%, 93.8%, 0.78%, and 1.43%, respectively. **g**–**i** In-vivo localization of MSCs in the small intestine 24 h after the establishment of the rat ANP model; the picture of the rat small intestine frozen section with (**g**) or without (**h**) fluorescence. The merged picture (**i**) with **g** and **h** showed that most of the MSCs localized to the small intestine mucous layer (×100)

The MSCs maintained strong proliferative ability and attached to the wall of the culture vessel. Passage 3 cells showed a spindle and fibroblast-like shape, and whirl-like distribution on culture plates (Fig. 1a). The cells were identified by their surface markers. High expression of CD 29/90 and low expression of CD 34/45 confirmed that these cells were MSCs (Fig. 1c–f). After CM-Dil labeling, red fluorescence was observed in the cytoplasm of MSCs (Fig. 1b). Small intestine frozen sections showed CM-Dil-labeled cells in the mucous layer of the 24-h MSC-treated rats (Fig. 1g–i).

### Amylase and albumin of blood and ascites

The ANP rats and the culture media-treated rats had a large amount of bloody ascites in the abdominal cavity. After treatment with MSCs, the output of ascites was decreased significantly at the corresponding time point. The results showed that the levels of serum amylase in the ANP group and the culture media-treated group were higher than the levels in the control group at the corresponding time point. With MSC treatment, the levels of serum and ascites amylase were lower than that of the ANP group and the culture media-treated group at the corresponding time point. The levels of serum albumin were negatively time-dependent in the ANP group and the culture media-treated group, and were lower than the control group at the corresponding time points. After treatment with MSCs, the levels of serum albumin were higher than levels observed in the ANP group and the culture media-treated group at the corresponding time points. The levels of ascites albumin were positively time-dependent in the ANP group and the culture media-treated group, and were higher than the levels in the MSC-treated group at the corresponding time points (Table 1).

**Table 1:** Results of amylase, albumin, ascites output, and Evans blue assays (*n* = 10 for each time point)

	6 h	12 h	24 h
Control group			
Serum amylase (IU/L)	834.10 ± 30.67	824.00 ± 20.50	831.10 ± 29.49
Serum albumin (g/L)	15.80 ± 0.29	15.60 ± 0.27	16.20 ± 0.92
Evans blue (ng/mg)	5.07 ± 0.47	5.62 ± 0.78	4.90 ± 0.56
ANP group			
Serum amylase (IU/L)	3831.30 ± 178.52*	6650.90 ± 366.15*	2470.70 ± 126.41*
Serum albumin (g/L)	13.10 ± 0.31*	11.40 ± 0.22*	6.90 ± 0.53*
Evans blue (ng/mg)	13.40 ± 0.97*	21.88 ± 1.19*	39.46 ± 1.48*^,^**
Ascites output^a^ (mL)	7.75 ± 0.84	13.35 ± 1.06	21.15 ± 1.69
Ascites albumin^a^ (mg)	46.00 ± 5.40	56.00 ± 5.20	88.00 ± 9.50
Ascites amylase^a^ (IU/L)	18,110.90 ± 752.26	33,017.70 ± 4605.52	11,582.70 ± 1040.69
Culture media-treated group			
Serum amylase (IU/L)	3898.30 ± 139.81*	6604.80 ± 356.46*	2456.00 ± 106.70*
Serum albumin (g/L)	13.03 ± 0.18*	11.48 ± 0.19*	6.94 ± 0.41*
Evans blue (ng/mg)	13.45 ± 0.16*	22.00 ± 0.53*	40.06 ± 1.27*
Ascites output^a^ (mL)	7.95 ± 0.84	13.90 ± 0.46	21.00 ± 1.21
Ascites albumin^a^ (mg)	45.80 ± 5.01	57.70 ± 1.62	88.90 ± 6.04
Ascites amylase^a^ (IU/L)	18,162.30 ± 584.23	33,064.80 ± 3960.86	11,590.10 ± 317.65
MSC-treated group			
Serum amylase (IU/L)	3179.80 ± 217.49^***^^,****^	5241.50 ± 399.00^***^^,****^	2038.30 ± 98.46^***^^,****^
Serum albumin (g/L)	13.88 ± 0.12^***^^,****^	12.19 ± 0.19^***^^,****^	9.48 ± 0.80^***^^,****^
Evans blue (ng/mg)	11.40 ± 0.77	18.62 ± 0.94^***^^,****^	33.22 ± 1.79^***^^,****^
Ascites output (mL)	7.06 ± 0.78	10.31 ± 0.93^***^^,****^	15.80 ± 1.44^***^^,****^
Ascites albumin (mg)	41.80 ± 5.40	43.30 ± 2.08^***^^,****^	61.00 ± 7.05^***^^,****^
Ascites amylase (IU/L)	15,296.00 ± 920.21^***^^,****^	19,558.00 ± 1627.54^***^^,****^	9097.90 ± 540.51^***^^,****^

^a^There was no ascites fluid observed in the control group

After induction of acute necrotizing pancreatitis (ANP) and treatment with mesenchymal stem cells (MSCs), serum and ascites were collected at different time points and assayed for amylase and albumin. Data are shown as mean ± SE

*p < 0.01, versus control group at the same time points; **p < 0.01 versus all other ANP group time points; ^***^ *p* < 0.05, versus ANP group at the same time points; ^****^ *p* < 0.05, versus culture media-treated group at the same time points; as tested using a two-sample *t* test, and one-way ANOVA for comparing several groups

### Histopathology of the pancreas

**Fig. 2:**
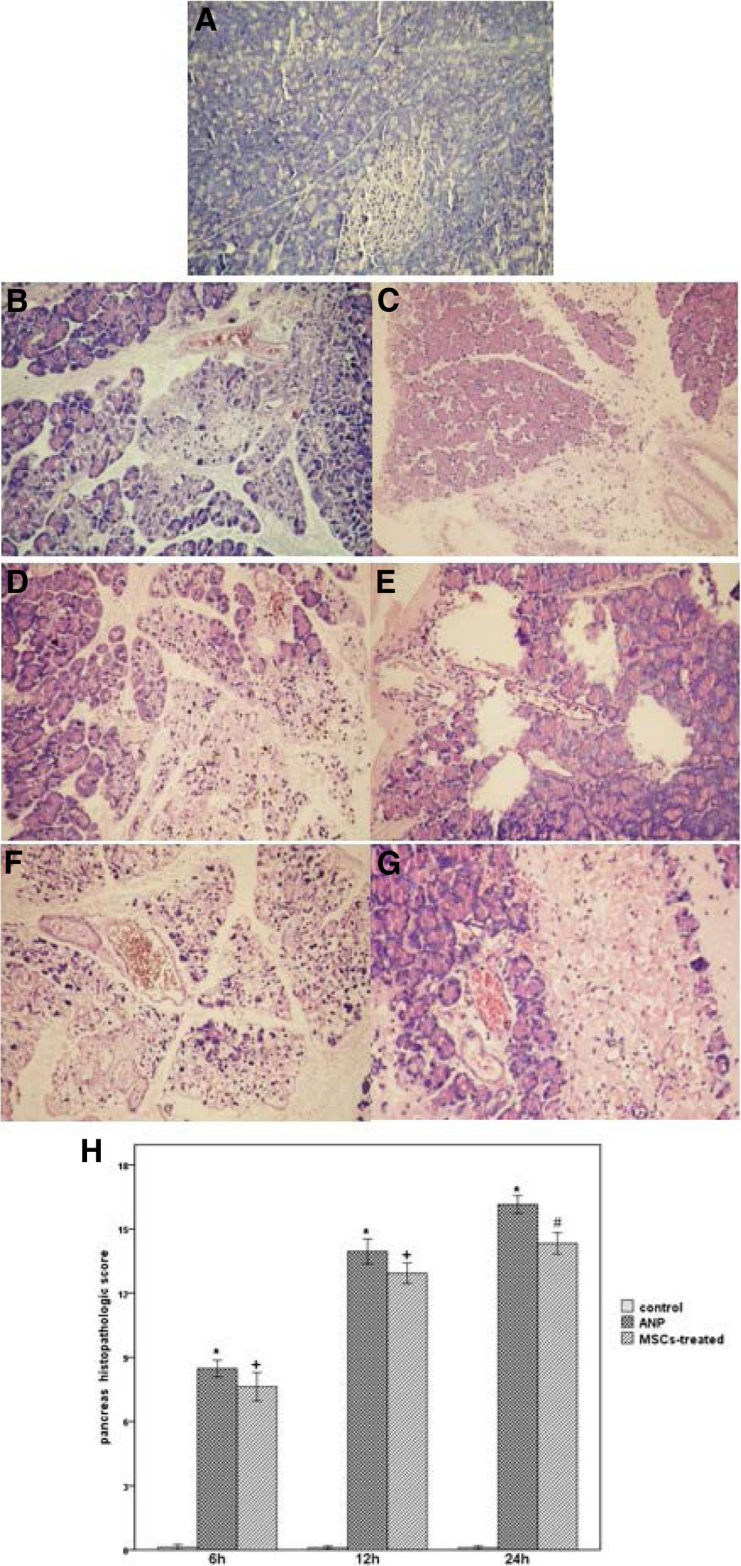
Histopathology of the pancreas. **a**–**g** H&E staining of rat pancreas (×100). There were no significant pathological changes in pancreatic tissues in the control group (**a**). The pancreas exhibited mild interstitial edema and a few inflammatory cells infiltrated in the acute necrotizing pancreatitis (*ANP*) group at 6 h (**b**). Compared to the ANP group at 12 h (**d**), the edema formation, hemorrhage, inflammatory cell infiltration, and necrosis was more severe in the ANP group at 24 h (**f**). Pancreatic damage was reduced significantly in the mesenchymal stem cells (*MSCs*)-treated group at the 6-h, 12-h, and 24-h time points (**c**, **e**, and **g**). **h** The pancreatic histopathologic score of each group is shown as the mean ± SE (*n* = 10). *p < 0.01, versus the control group at the same time point; ^+^ *p* < 0.05, ^#^ *p* < 0.01, versus the ANP group at the same time point; as tested using a two-sample *t* test for comparing between two groups, and one-way ANOVA for comparing several groups

The ANP model was established successfully. Rats had visible saponification spots on the omentum and mesentery. Under light microscopy, there were no significant pathological changes in pancreatic tissues in rats in the control group. However, the pancreatic damage in the ANP group was more severe than that in the control group at the corresponding time points. After treatment with MSCs, pathological changes were milder than changes observed in the ANP group (Fig. 2).

### Small intestine capillary leakage

**Fig. 3:**
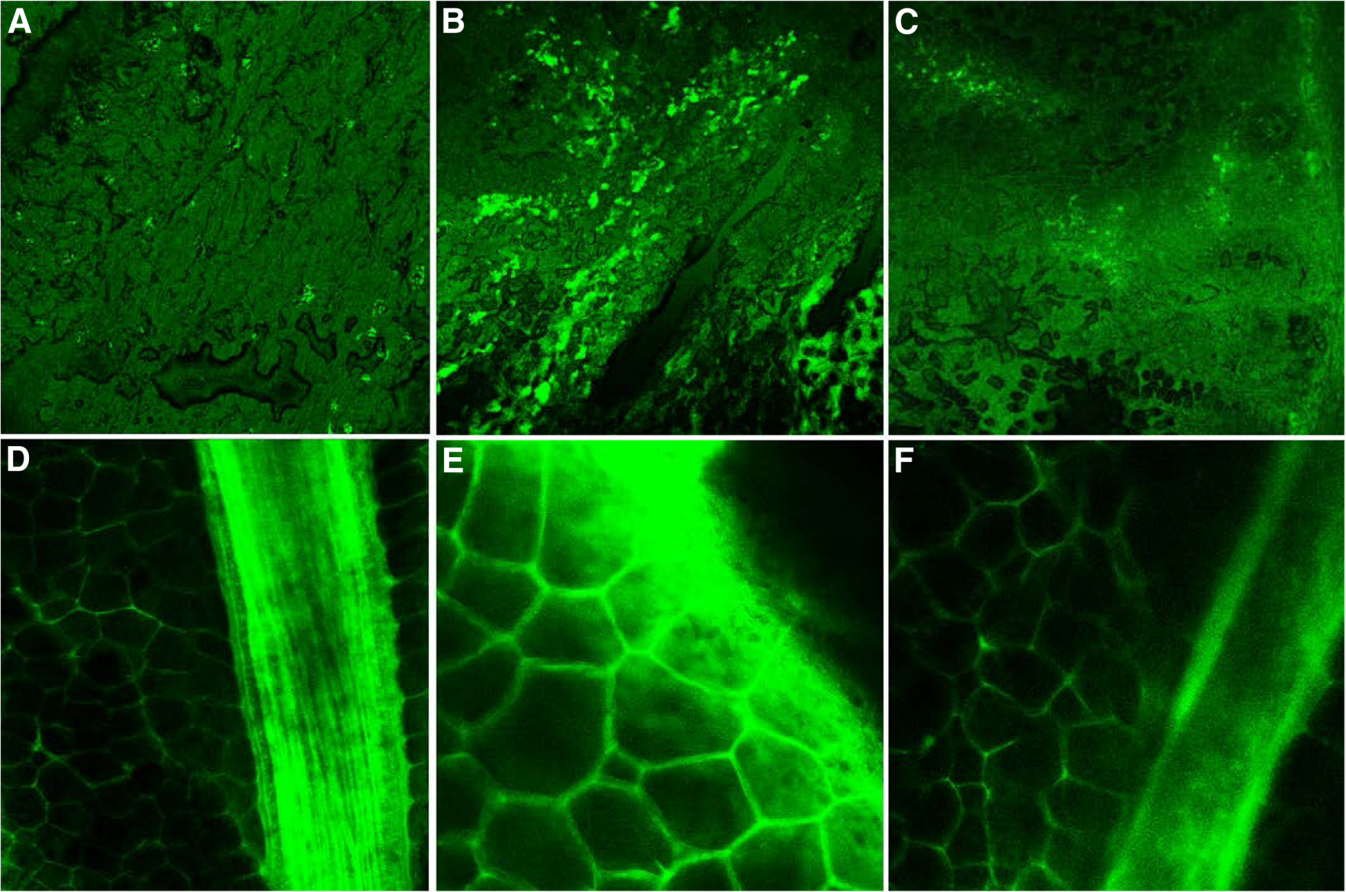
Tracking of infused FITC-albumin in the small intestine and the mesentery of the small intestine 24 h after the establishment of the rat ANP model. **a**–**c** Small intestine frozen sections (×50). FITC-albumin located in the small intestinal capillaries in the control group (**a**). Compared to the control group, a lot of extravasated FITC-albumin was seen in the intestinal tissues in the ANP group (**b**). After treatment with MSCs, extravasation of the FITC-albumin was less than that in the ANP group (**c**). **d**–**f** Small intestinal mesentery covered glass slide (×50). FITC-albumin located in the small intestine mesentery capillaries in the control group (**d**). High levels of FITC-albumin were observed in the areas of perivascular space in the ANP group (**e**). Extravasation of the FITC-albumin was reduced significantly in the MSC-treated group (**f**)

Small intestine capillary leakage caused by ANP was evaluated through the Evans blue leakage assay. The levels of Evans blue content in intestinal tissues were positively time-dependent in the ANP and the culture media-treated groups, and were higher than the control group at the corresponding time points. After treatment with MSCs, the levels of Evans blue content in intestinal tissues were lower than the levels in the ANP group and the culture media-treated group at the corresponding time points (Table 1). The small intestine frozen sections and small intestinal mesentery covered glass slide showed increased amounts of FITC-albumin which had leaked out of the capillary in the ANP group compared to the control group. After treatment with MSCs, the leakage of FITC-albumin decreased significantly at the corresponding time points (Fig. 3).

### Endothelial gap morphology demonstrated by SEM

**Fig. 4:**
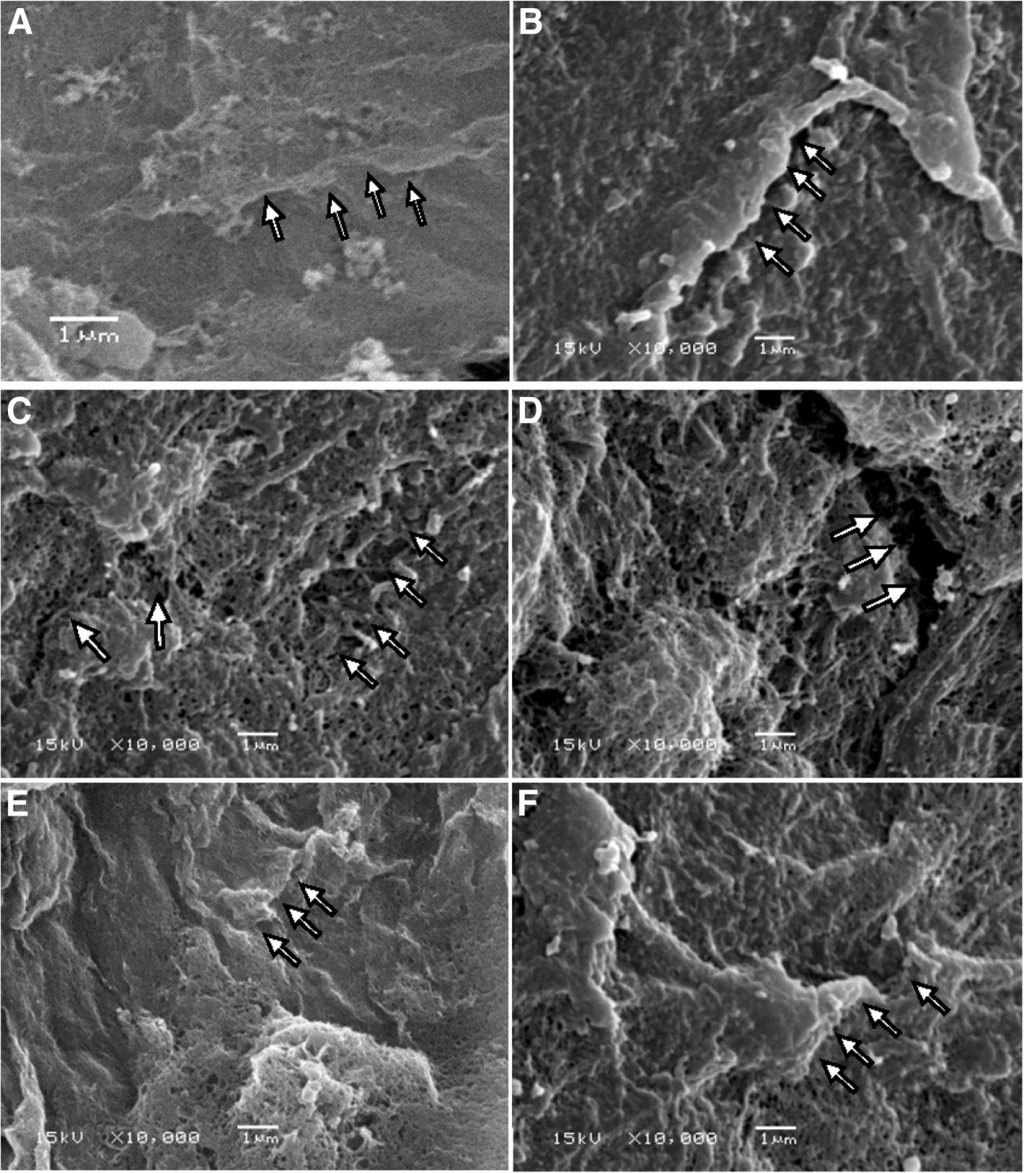
Scanning electron microscopy of endothelium in venules of rat small intestine mesentery. **a** The luminal surface of the small intestine mesentery venules was smooth and flat in the control group. **b**–**d** The damage to the connections of venule endothelial cells increased over time after induction of ANP (**b**, ANP 6 h; **c**, ANP 12 h; **d**, ANP 24 h). The gaps between connections of endothelial cells at 24 h was the most wide of all time points (**d**). **e**–**f** After treatment with MSCs, the damage was less than that observed in the ANP group at the corresponding time points (**e**, MSC-treated 12 h; **f**, MSC-treated 24 h). The arrows refer to the connections of venule endothelial cells

The change of the luminal surface of small intestinal mesentery venules was observed using SEM. The damage to the connections of venule endothelial cells increased over time after induction of ANP. The gaps between connections of endothelial cells progressed at each time point, and were most noticeable at the 24-h time point. After treatment with MSCs, the damage observed was less than that seen in the ANP group at the corresponding time points (Fig. 4).

### AQP1 expression in intestinal tissues

**Fig. 5:**
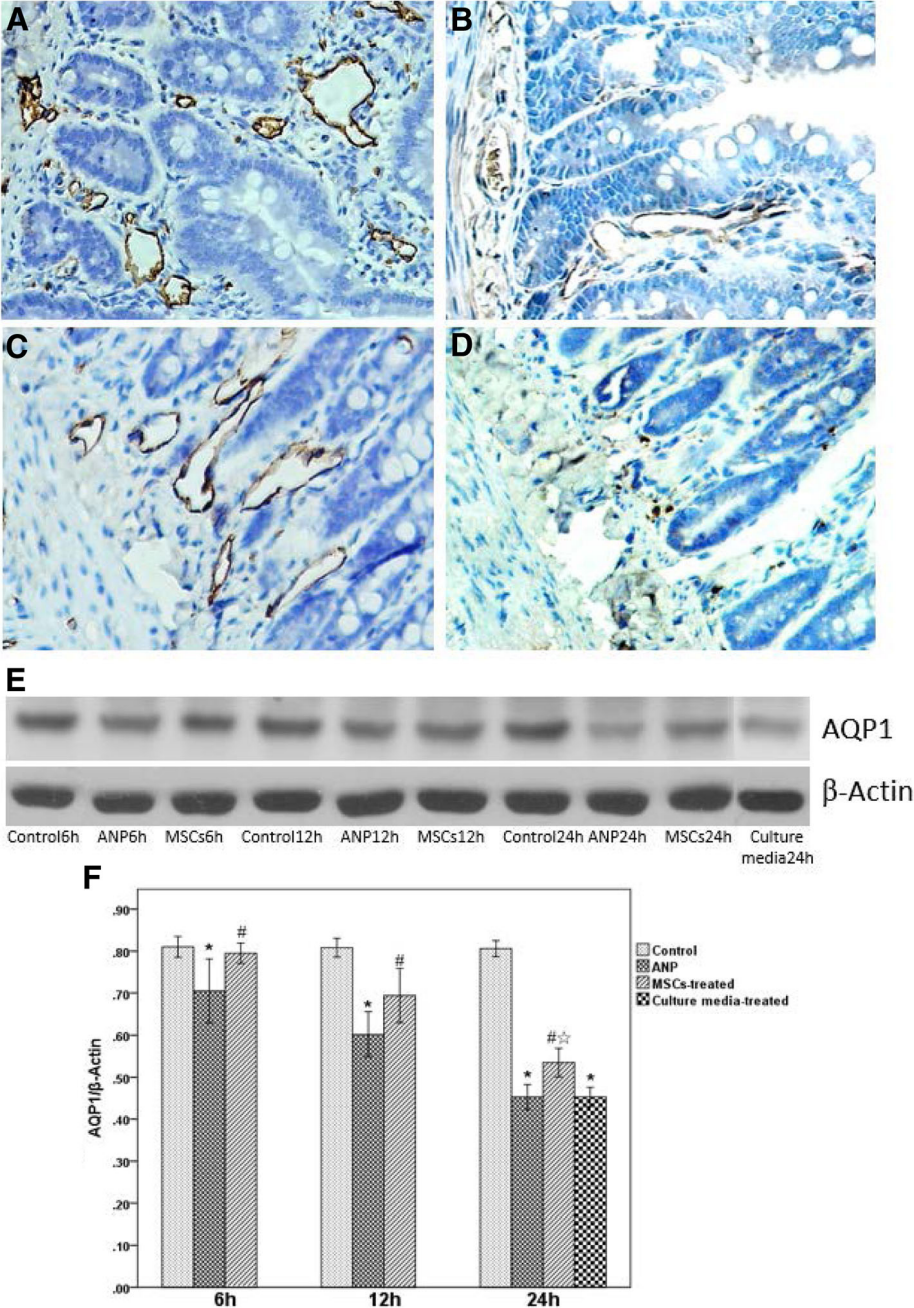
Intestinal aquaporin 1 (*AQP1*) expression. **a**–**d** Immunohistochemistry of AQP1 (×100). There was high expression of AQP1 on the plasma membrane of the endothelial cells in the intestinal capillary in the control group (**a**). Compared with the control group, the expression of AQP1 decreased significantly in the acute necrotizing pancreatitis (*ANP*) group at 24 h (**b**). After treatment with mesenchymal stem cells (*MSCs*), the expression of AQP1 was higher than the expression in the ANP group at 24 h (**c**). Compared with the ANP group at 24 h, the expression of AQP1 was not significantly changed after treatment with culture media (**d**). **e**, **f** Western blotting of AQP1 protein in the small intestine. Values are presented as the mean ± SE (*n* = 10). *p < 0.01, versus the control group at 24 h and all ANP group time points; ^#^ *p* < 0.05, versus the ANP group at the same time point; ^☆^ *p* < 0.05, versus the culture media-treated group at 24 h; as tested using a two-sample *t* test for comparing between two groups, and one-way ANOVA for comparing several groups

The result of immunohistochemistry (IHC) staining showed that AQP1 was expressed predominantly on the plasma membrane of the endothelial cells and the erythrocytes in the vessel (Fig. 5a). Compared with the control group, the expression of AQP1 decreased significantly at 24 h after induction of ANP (Fig. 5b). After treatment with MSCs, the expression of AQP1 was higher than the ANP group at the corresponding time point using IHC (Fig. 5c) and Western blotting (Fig. 5d and e), respectively.

Real-time PCR analysis showed that AQP1 mRNA expression levels in intestinal tissues were negatively time-dependent in the ANP group and the culture media-treated group, and were lower than levels observed in the control group at the corresponding time points. Compared to the ANP group and the culture media-treated group, the AQP1 mRNA expression levels were higher in the MSC-treated group at the corresponding time points (Table 2).

**Table 2:** Small intestine aquaporin 1 mRNA expression

2^–∆∆CT^	6 h	12 h	24 h
Control group	1.00 ± 0.02	0.99 ± 0.02	1.00 ± 0.01
ANP group	0.77 ± 0.01*	0.54 ± 0.02*	0.40 ± 0.02*^,^**
Culture media-treated group	0.78 ± 0.02*	0.53 ± 0.01*	0.39 ± 0.01*^,^**
MSC-treated group	0.84 ± 0.01^***^^,****^	0.60 ± 0.03^***^^,****^	0.47 ± 0.03^***^^,****^

After induction of acute necrotizing pancreatitis (ANP) and treatment with mesenchymal stem cells (MSCs), quantitative real-time polymerase chain reaction was used to estimate changes in the expression of small intestine mRNA

*p < 0.01, versus control group at the same time points; **p < 0.01, versus the ANP group at all time points; ^***^ *p* < 0.05, versus the ANP group at the same time points; ^****^ *p* < 0.05, versus the culture media-treated group at the same time points; as tested using a two-sample *t* test, and one-way ANOVA for comparing several groups

## Discussion

The cascaded release of various inflammatory mediators and cytokines during the early stages of ANP may damage the capillary endothelial barrier and lead to capillary leakage. At this time, a lot of intravascular contents leak into the third space, which may lead to the reduction of effective circulatory blood volume and the insufficient end-organ perfusion that could induce multiple organ dysfunction [28, 29]. Capillary leakage in the lung leads to interstitial edema and alveolar fluid accumulation, which is seen during acute lung injury and respiratory distress syndrome in ANP [29, 30]. Retroperitoneal edema, fluid collections, ascites, and intestinal edema occurs in the abdomen during ANP, leading to intra-abdominal hypertension. This can contribute to insufficient end-organ perfusion within the cardiovascular, respiratory, and renal systems [31].

Large volumes of fluid accumulate in the peritoneal cavity in ANP. Ascites fluid can induce inflammatory cytokines and enhance the inflammatory response associated with severe acute pancreatitis [32, 33], and can play important roles in the pathologic course of this disease [34]. The rapid rise in the amount of ascites output and the levels of amylase and albumin in the blood and ascites are characteristic, and are usually diagnostic criteria of acute pancreatitis [35–37]. In our study, we found an increased amount of ascites, elevated ascitic albumin, and decreased serum albumin in rats with ANP over time, indicating that leakage of abdominal organs gradually increased with disease progression. Gut permeability was correlated with the severity of acute pancreatitis [38]. The Evans blue assay and tracking of FITC-albumin indicated that there was significantly increased intestinal capillary leakage in the ANP rats compared with the control group. Gradually increasing leakage was observed in the ANP rat model at 6 h, 12 h and 24 h. Using SEM, the damage to the connections of endothelial cells of venules was increased over time after induction of ANP. The gaps between connections of endothelial cells progressed at each time point, and were most noticeable at the 24-h time point.

AQPs are integral hydrophobic membrane proteins that could form the water channel and facilitate water movement across the cell membrane. There are 13 different AQPs in the AQP family that exist in various tissues [2]. AQP1 was the first member of the AQP family to be identified in erythrocytes [3]. In the small intestine, AQP1 is widely distributed on the capillary or microvascular endothelium of lymphatic cells [4–7]. Studies have shown that abnormalities in AQP1 contribute to the development of many diseases [2, 6]. The expression of AQP1 was significantly reduced in a murine model of LPS-induced acute lung injury, and in the pancreas, lung, and intestinal tissue of acute necrotizing pancreatitis in rats [9]. Our results show that the expression of AQP1 in the small intestine decreased gradually over time in ANP, accompanied by a time-dependent increase in leakage.

The capillary endothelial barrier consists of capillary endothelial cells, connections between endothelial cells, and the basement membrane. Any changes in these three components may lead to changes in capillary permeability and leakage. The damage to the connections of capillary endothelial cells increased over time after the induction of ANP. The gaps between connections of endothelial cells caused damage to the intestinal capillary endothelial barrier, resulting in a large amount of bloody ascites in the abdominal cavity in the ANP group. AQP1 can mediate inter-cytomembrane water transportation, resulting in the transport of water extravasated to interstitial tissue or the abdominal cavity back into the vessels across the capillary wall. This results in reduced tissue edema and ascites [3, 39, 40]. The expression of AQP1 decreased significantly after induction of ANP. This induced edema within the small intestine, resulting in the impairment of small intestinal microcirculation and injury to the small intestine in ANP.

Studies have shown that MSCs have strong differentiation potential. There are few ethical issues with their use. Additionally, they are easy to access, have weak immunogenicity, and are able to regulate the immune system and regenerate damaged tissue. Taken together, MSCs are good candidates for cell-based therapy [13, 41]. MSCs could repair injured alveolar epithelium induced by LPS in mice [42] and reduced lung histologic damage in septic mice [43]. The results of pancreatic pathological damage are consistent with prior research [14]. MSCs could reduce inflammation and damage to pancreatic tissue in the rat model of acute pancreatitis. MSC treatment resulted in faster recovery to the structure and function of the small intestine in the mouse model of radiation-induced gastrointestinal tract damage [15, 23]. MSCs could differentiate into vascular endothelial cells and improve cardiac function in a rat model of dilated cardiomyopathy [11, 44, 45].

Three different MSC delivery routes (intravenous, intraperitoneal, and anal injection) were used in the studies of intestinal disease [15, 23, 24, 46–48]. For the reasons that follow, MSCs were transplanted via tail vein injection in this study. 1) Anal injections are usually used to study large intestinal diseases [49]. 2) The intravenous injection is historically the most common method for MSC delivery [15, 17, 23, 24, 47, 48, 50] and was identified to be better in comparison with intraperitoneal injection [51]. 3) Large volumes of ascites fluid accumulate in the peritoneal cavity in ANP. 4) The complications associated with intraperitoneal injection are a concern. These complications include that stem cells may irritate the peritoneal lining causing peritonitis [52] and physical damage to intra-abdominal structures, such as vaginal vault perforation, bladder erosion, and bowel perforation [53]. 5) Structural changes to the capillary endothelial barrier are one of the objectives in this study. MSCs that are injected intravenously may be more effective for the repair of damaged capillary endothelial cells.

Similar to previous studies [15, 23, 24], MSCs might reside in the small intestine. In this study the amount of CM-Dil-labeled MSCs was observed under a confocal microscope. After the treatment with MSCs, the serum amylase, ascitic amylase, ascites output, and ascitic albumin were decreased significantly, and the serum albumin was increased. We found that the level of small intestinal capillary leakage was alleviated, and the damage to the pancreas and small intestine was ameliorated. Furthermore, the expression of small intestinal AQP1 was enhanced after MSC therapy. These parameters were not significantly altered after treatment with culture media, excluding culture medium as a factor in the MSC-treated group. The MSCs may exert their therapeutic effects on differentiation and replacement of the damaged capillary endothelial cells. Alternatively, they may influence cell-to-cell contact, or interact with immune cells [46], diminishing the various inflammatory mediators and cytokines which are normally released during ANP.

## Conclusions

The damage to the intestinal capillary endothelial barrier and the dysfunction of AQP1 could lead to capillary leakage in ANP. MSCs could decrease small intestinal capillary leakage and microcirculation disorder by diminishing the damage to the capillary endothelial barrier and reversing the decease of AQP1 expression in the small intestine. MSCs lessen small intestinal injury in rats with ANP.

## Abbreviations

ANP: Acute necrotizing pancreatitis
AQP: Aquaporin
DMEM: Dulbecco’s modified Eagle’s medium
FCS: Fetal calf serum
FITC: Fluorescein isothiocyanate
H&E: Hematoxylin and eosin
IHC: Immunohistochemistry
LPS: Lipopolysaccharide
MSC: Mesenchymal stem cell
PBS: Phosphate-buffered saline
PCR: Polymerase chain reaction
SD: Sprague–Dawley
SEM: Scanning electron microscopy

## References

[1] Bassi C, Falconi M, Butturini G, Pederzoli P, Holzheimer RG, Mannick JA (2001). Surgical treatment: evidence-based and problem-oriented.

[2] Verkman AS (2002). Physiological importance of aquaporin water channels. Ann Med.

[3] Agre P, Preston GM, Smith BL (1993). Aquaporin CHIP: the archetypal molecular water channel. Am J Physiol.

[4] Takata K, Matsuzaki T, Tajika Y (2004). Aquaporins: water channel proteins of the cell membrane. Prog Histochem Cytochem.

[5] Tsujikawa T, Itoh A, Fukunaga T, Satoh J, Yasuoka T, Fujiyama Y (2003). Alteration of aquaporin mRNA expression after small bowel resection in the rat residual ileum and colon. J Gastroenterol Hepatol.

[6] Nagahama M, Ma N, Semba R, Naruse S (2006). Aquaporin 1 immunoreactive enteric neurons in the rat ileum. Neurosci Lett.

[7] Laforenza U (2012). Water channel proteins in the gastrointestinal tract. Mol Aspects Med.

[8] Su X, Song Y, Jiang J, Bai C (2004). The role of aquaporin-1 (AQP1) expression in a murine model of lipopolysaccharide-induced acute lung injury. Respir Physiol Neurobiol.

[9] Feng DX, Peng W, Chen YF (2012). Down-regulation of aquaporin 1 in rats with experimental acute necrotizing pancreatitis. Pancreas.

[10] Dawn B, Bolli R (2005). Adult bone marrow-derived cells: regenerative potential, plasticity, and tissue commitment. Basic Res Cardiol.

[11] Nagaya N, Kangawa K, Itoh T (2005). Transplantation of mesenchymal stem cells improves cardiac function in a rat model of dilated cardiomyopathy. Circulation.

[12] Vieyra DS, Jackson KA, Goodell MA (2005). Plasticity and tissue regenerative potential of bone marrow-derived cells. Stem Cell Rev.

[13] Rojas M, Xu J, Woods CR (2005). Bone marrow-derived mesenchymal stem cells in repair of the injured lung. Am J Respir Cell Mol Biol.

[14] Jung KH, Song SU, Yi T (2011). Human bone marrow-derived clonal mesenchymal stem cells inhibit inflammation and reduce acute pancreatitis in rats. Gastroenterology.

[15] Semont A, Mouiseddine M, Francois A (2010). Mesenchymal stem cells improve small intestinal integrity through regulation of endogenous epithelial cell homeostasis. Cell Death Differ.

[16] Chen QH, Liu AR, Qiu HB, Yang Y (2015). Interaction between mesenchymal stem cells and endothelial cells restores endothelial permeability via paracrine hepatocyte growth factor in vitro. Stem Cell Res Ther.

[17] Jin M, Chen Y, Zhou Y (2016). Transplantation of bone marrow-derived mesenchymal stem cells expressing elastin alleviates pelvic floor dysfunction. Stem Cell Res Ther.

[18] Jackson JS, Golding JP, Chapon C, Jones WA, Bhakoo KK (2010). Homing of stem cells to sites of inflammatory brain injury after intracerebral and intravenous administration: a longitudinal imaging study. Stem Cell Res Ther.

[19] Zhao W, Li JJ, Cao DY (2012). Intravenous injection of mesenchymal stem cells is effective in treating liver fibrosis. World J Gastroenterol.

[20] Schmidt J, Rattner DW, Lewandrowski K (1992). A better model of acute pancreatitis for evaluating therapy. Ann Surg.

[21] Aho HJ, Koskensalo SM, Nevalainen TJ (1980). Experimental pancreatitis in the rat. Sodium taurocholate-induced acute haemorrhagic pancreatitis. Scand J Gastroenterol.

[22] Liu HB, Cui NQ, Wang Q, Li DH, Xue XP (2008). Sphingosine-1-phosphate and its analogue FTY720 diminish acute pulmonary injury in rats with acute necrotizing pancreatitis. Pancreas.

[23] Kudo K, Liu Y, Takahashi K (2010). Transplantation of mesenchymal stem cells to prevent radiation-induced intestinal injury in mice. J Radiat Res.

[24] Tu XH, Song JX, Xue XJ (2012). Role of bone marrow-derived mesenchymal stem cells in a rat model of severe acute pancreatitis. World J Gastroenterol.

[25] Cui L, Takagi Y, Wasa M, Sando K, Khan J, Okada A (1999). Nitric oxide synthase inhibitor attenuates intestinal damage induced by zinc deficiency in rats. J Nutr.

[26] Kraneveld AD, Buckley TL, van Heuven-Nolsen D, van Schaik Y, Koster AS, Nijkamp FP (1995). Delayed-type hypersensitivity-induced increase in vascular permeability in the mouse small intestine: inhibition by depletion of sensory neuropeptides and NK1 receptor blockade. Br J Pharmacol.

[27] Lange S, Delbro DS, Jennische E (1994). Evans blue permeation of intestinal mucosa in the rat. Scand J Gastroenterol.

[28] Eibl G, Hotz HG, Faulhaber J, Kirchengast M, Buhr HJ, Foitzik T (2000). Effect of endothelin and endothelin receptor blockade on capillary permeability in experimental pancreatitis. Gut.

[29] Cappell MS (2008). Acute pancreatitis: etiology, clinical presentation, diagnosis, and therapy. Med Clin North Am.

[30] Talamini G, Uomo G, Pezzilli R (1999). Serum creatinine and chest radiographs in the early assessment of acute pancreatitis. Am J Surg.

[31] De Waele JJ, Leppaniemi AK (2009). Intra-abdominal hypertension in acute pancreatitis. World J Surg.

[32] Denham W, Yang J, Fink G, Zervos EE, Carter G, Norman J (1997). Pancreatic ascites as a powerful inducer of inflammatory cytokines. The role of known vs unknown factors. Arch Surg.

[33] Gou S, Yang C, Yin T (2015). Percutaneous catheter drainage of pancreatitis-associated ascitic fluid in early-stage severe acute pancreatitis. Pancreas.

[34] Satoh A, Shimosegawa T, Masamune A, Fujita M, Koizumi M, Toyota T (1999). Ascitic fluid of experimental severe acute pancreatitis modulates the function of peritoneal macrophages. Pancreas.

[35] Koizumi M, Takada T, Kawarada Y (2006). JPN Guidelines for the management of acute pancreatitis: diagnostic criteria for acute pancreatitis. J Hepatobiliary Pancreat Surg.

[36] McMahon MJ, Playforth MJ, Pickford IR (1980). A comparative study of methods for the prediction of severity of attacks of acute pancreatitis. Br J Surg.

[37] Mayer AD, Airey M, Hodgson J, McMahon MJ (1985). Enzyme transfer from pancreas to plasma during acute pancreatitis. The contribution of ascitic fluid and lymphatic drainage of the pancreas. Gut.

[38] Ryan CM, Schmidt J, Lewandrowski K (1993). Gut macromolecular permeability in pancreatitis correlates with severity of disease in rats. Gastroenterology.

[39] Ma T, Verkman AS (1999). Aquaporin water channels in gastrointestinal physiology. J Physiol.

[40] Benga G (2003). Birth of water channel proteins—the aquaporins. Cell Biol Int.

[41] Bartholomew A, Sturgeon C, Siatskas M (2002). Mesenchymal stem cells suppress lymphocyte proliferation in vitro and prolong skin graft survival in vivo. Exp Hematol.

[42] Cai SX, Liu AR, Chen S (2015). Activation of Wnt/β-catenin signalling promotes mesenchymal stem cells to repair injured alveolar epithelium induced by lipopolysaccharide in mice. Stem Cell Res Ther.

[43] Güldner A, Maron-Gutierrez T, Abreu SC (2015). Expanded endothelial progenitor cells mitigate lung injury in septic mice. Stem Cell Res Ther.

[44] Wang XJ, Li QP (2007). The roles of mesenchymal stem cells (MSCs) therapy in ischemic heart diseases. Biochem Biophys Res Commun.

[45] Pati S, Khakoo AY, Zhao J (2011). Human mesenchymal stem cells inhibit vascular permeability by modulating vascular endothelial cadherin/beta-catenin signaling. Stem Cells Dev.

[46] Wang M, Liang C, Hu H (2016). Intraperitoneal injection (IP), intravenous injection (IV) or anal injection (AI)? Best way for mesenchymal stem cells transplantation for colitis. Sci Rep.

[47] González MA, Gonzalez-Rey E, Rico L, Büscher D, Delgado M (2009). Adipose-derived mesenchymal stem cells alleviate experimental colitis by inhibiting inflammatory and autoimmune responses. Gastroenterology.

[48] Chen Z, He X, He X (2014). Bone marrow mesenchymal stem cells ameliorate colitis-associated tumorigenesis in mice. Biochem Biophys Res Commun.

[49] Robinson AM, Sakkal S, Park A (2014). Mesenchymal stem cells and conditioned medium avert enteric neuropathy and colon dysfunction in guinea pig TNBS-induced colitis. Am J Physiol Gastrointest Liver Physiol.

[50] Kean TJ, Lin P, Caplan AI, Dennis JE (2013). MSCs: delivery routes and engraftment, cell-targeting strategies, and immune modulation. Stem Cells Int.

[51] Gonçalves FC, Schneider N, Pinto FO (2014). Intravenous vs intraperitoneal mesenchymal stem cells administration: what is the best route for treating experimental colitis. World J Gastroenterol.

[52] Liu ZC, Chang TM (2012). Intrasplenic transplantation of bioencapsulated mesenchymal stem cells improves the recovery rates of 90% partial hepatectomized rats. Stem Cells Int.

[53] Makhija S, Leitao M, Sabbatini P (2001). Complications associated with intraperitoneal chemotherapy catheters. Gynecol Oncol.

